# Apnea during moderate to deep sedation using continuous infusion of remimazolam compared to propofol and dexmedetomidine: A retrospective observational study

**DOI:** 10.1371/journal.pone.0301635

**Published:** 2024-04-17

**Authors:** Chahyun Oh, Jiyong Lee, Jieun Lee, Yumin Jo, Sanghun Kwon, Minhae Bang, Chaeseong Lim, Yoon-Hee Kim, Sun Yeul Lee, Boohwi Hong

**Affiliations:** 1 Department of Anesthesiology and Pain Medicine, Chungnam National University Hospital, Daejeon, Korea; 2 Department of Anesthesiology and Pain Medicine, College of Medicine, Chungnam National University, Daejeon, Korea; 3 Big Data Center, Biomedical Research Institute, Chungnam National University Hospital, Daejeon, Korea; Scuola Superiore Sant’Anna, ITALY

## Abstract

Remimazolam’s rapid onset and offset make it an innovative sedative for use during regional anesthesia. However, its respiratory safety profile is not well understood. We compared the continuous infusion of remimazolam with commonly used sedatives, propofol and dexmedetomidine, after regional anesthesia. In this retrospective study, the incidence of apnea (>10 seconds) was assessed in patients who underwent orthopedic surgery under regional anesthesia and received moderate to deep sedation using continuous infusion of remimazolam (group R: 0.1 mg/kg in 2 minutes followed by 0.5 mg/kg/hr). The incidence was compared with that of propofol (group P: 2–3 μg/mL target-controlled infusion) and dexmedetomidine (group D: 1 μg/kg in 10 minutes followed by 0.4–1 μg/kg/hr). Propensity score weighted multivariable logistic regression model was utilized to determine the effects of the sedative agents on the incidence of apnea. A total of 634 (191, 278, and 165 in group R, P, and D) cases were included in the final analysis. The incidence of apnea was 63.9%, 67.3%, and 48.5% in group R, P, and D, respectively. The adjusted odds ratios for apnea were 2.33 (95% CI, 1.50 to 3.61) and 2.50 (95% CI, 1.63 to 3.85) in group R and P, compared to group D. The incidence of apnea in patients receiving moderate to deep sedation using continuous infusion of remimazolam with dosage suggested in the current study was over 60%. Therefore, careful titration and respiratory monitoring is warranted.

## Introduction

Sedation not only enables a physician to maintain stable performance during the procedure but also provides comfort to a patient. It is therefore widely used in numerous areas of clinical practice. However, almost all hypnotics have cardiorespiratory risks accompanied by loss of consciousness. Therefore, a thorough understanding and careful monitoring of those risks are crucial.

Propofol and dexmedetomidine have been widely used for sedation during various procedures, surgeries, or critical patient care. Propofol gained its popularity due to its fast on- and offset which is a favorable feature for brief or outpatient-based procedures. However, it can exert considerable hemodynamic and respiratory impact which warrants cautious dosing [1]. Meanwhile, dexmedetomidine has gained clinical interest due to its favorable respiratory [2, 3] and cognitive profile [4] and potential benefits for a reduced rate of delirium [5]. However, it can also negatively impact hemodynamics often manifested as bradycardia and hypotension [2].

Remimazolam, which was recently introduced in clinical practice, has a similar chemical structure and pharmacodynamic characteristics to midazolam, but has distinctive pharmacokinetics due to its carboxylic ester moiety which leads to rapid hydrolysis by non-specific tissue esterase [6]. This pharmacokinetic feature enables a clinician to achieve deeper levels of sedation as needed without sacrificing its favorable recovery profile. Being a newly introduced drug, however, there is still scarce clinical data regarding its respiratory safety profile during sedation via continuous infusion.

In this study, the incidence of apnea was assessed in patients who underwent orthopedic surgery under regional anesthesia and received moderate to deep sedation using continuous infusion of remimazolam. The incidence of apnea was then compared with that of the two conventional regimens: continuous infusion of propofol or dexmedetomidine. The apnea was retrospectively detected using intraoperative recordings of high-resolution physiological signal data and an automated apnea detection algorithm. In addition, we explored factors related to the occurrence of apnea during remimazolam use.

## Material and methods

### Study design

The study protocol was approved by the Chungnam National University Hospital Institutional Review Board (IRB number: CNUH 2022-02-078). The requirement for written informed consent was waived by the Chungnam National University Hospital Institutional Review Board. The study data was collected from April 1 to 8, 2022, and it does not contain any personal identification information about individual patients. This retrospective study included patients aged over 17, American Society of Anesthesiology (ASA) physical status 1 to 3, who underwent orthopedic surgery under regional anesthesia (neuraxial or brachial plexus blockade) and sedation with continuous infusion of dexmedetomidine, propofol, or remimazolam from February to December 2021. Patients were excluded if their intraoperative monitoring did not include expired carbon dioxide (CO_2_) monitoring or absence of entire record. Other data collected from patient records included patient characteristics (age, sex, weight, height, BMI [body mass index], ASA physical status, comorbidities), and other clinical characteristics (anesthesia type [brachial plexus block or spinal anesthesia], surgery date, duration of surgery, surgical position [supine or non-supine], sedative agent and dose, duration of sedative administration, and any supplementary drugs for sedation). Patients were designated as group R (remimazolam), P (propofol), or D (dexmedetomidine) according to the primary sedative used. This manuscript adheres to the applicable STROBE (Strengthening the Reporting of Observational Studies in Epidemiology) guidelines.

### Anesthesia protocol

**Fig 1:**
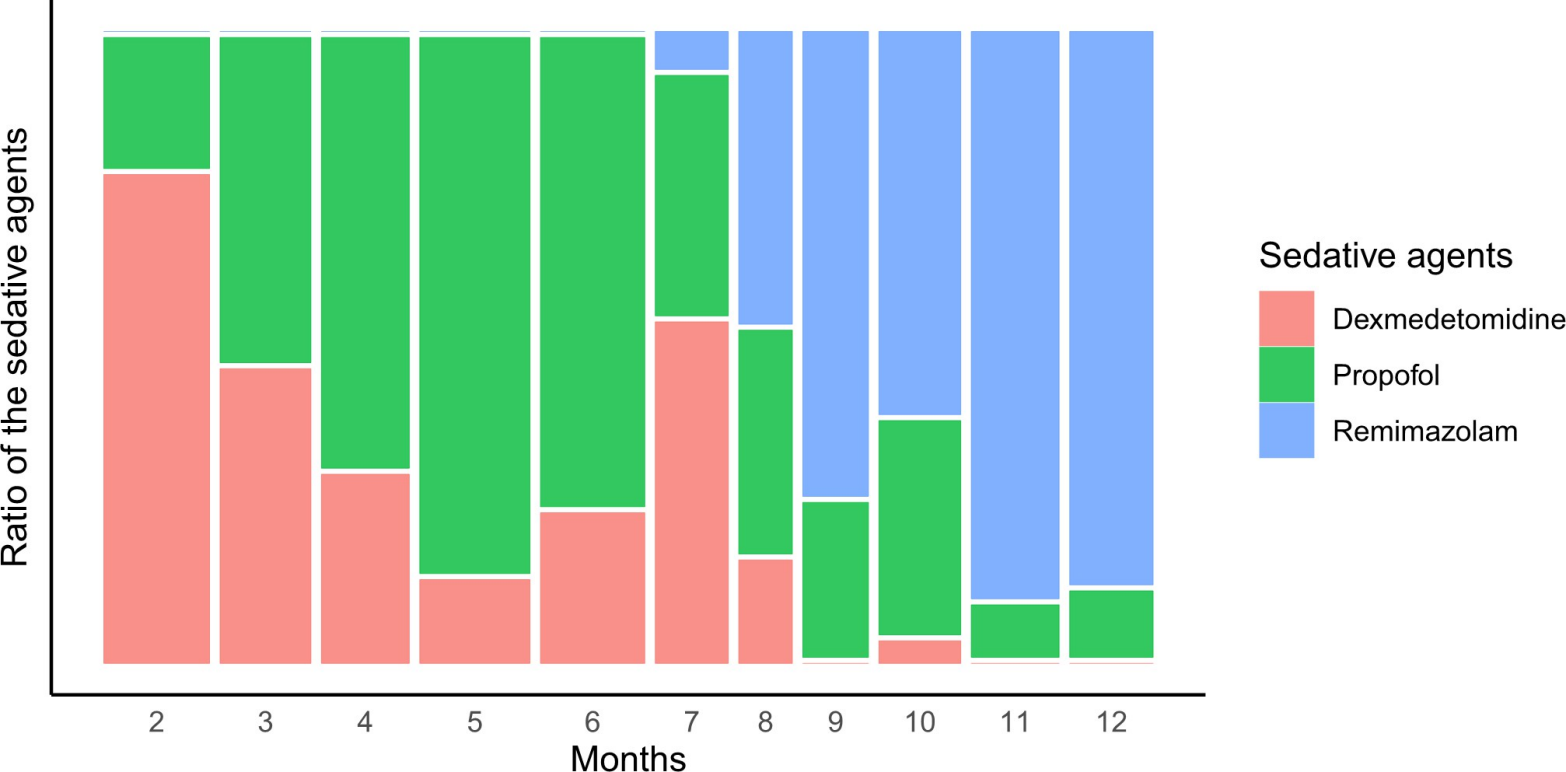
The trend of sedative uses during the study period.

All patients were sedated in the operating room after verifying adequate onset of the regional blockade in the surgical area. Sedative agents were selected according to the attending anesthesiologist’s preference. During the study period, there was a change in the preference of sedative use because of the introduction of remimazolam to our institution in July 2021 (Fig 1). Either of the three agents, remimazolam (Byfavo; Hana Pharmaceutical, Seoul, South. Korea), propofol (Fresofol MCT 2% Inj.; Fresenius Kabi Austria GmbH, Graz, Austria), or dexmedetomidine (Precedex Premix injection; Pfizer Pharmaceuticals Korea Ltd., Seoul, Korea) was continuously administered via syringe pump. The institutional protocol for these agents were as follows: (1) remimazolam: 3 mg/kg/hr over 2 minutes (0.1 mg/kg of loading dose) followed by 0.5 mg/kg/hr continuous infusion; (2) propofol: administered using target-controlled infusion (Schnider model), starting with 2–3 μg/mL; (3) dexmedetomidine: 1 μg/kg infused over 10 minutes followed by 0.4–1 μg/kg/hr continuous infusion. The doses for remimazolam and dexmedetomidine were based on ideal body weight. All dosages were titrated to achieve moderate to deep sedation (Richmond Agitation & Sedation Scale –3 to –4) under the discretion of the attending anesthesiologist. Supplementary drugs such as small bolus doses of propofol (20–30 mg), midazolam (1–3 mg), or fentanyl (20–40 μg) were allowed to relieve intolerable discomfort or to control persistent or sudden movement of the patient during the procedure. Supplementary oxygen was routinely administered via a simple facial mask at a rate of equal to or above 5 L/min with side port expired CO_2_ monitoring before the beginning of the sedation.

A trend of sedative uses was noted during the study period due to the introduction of remimazolam to the institution. The use of remimazolam was gradually increased from July 2021.

### Data acquisition

All physiological data were obtained from the prospective registry of vital signs for surgical patients at Chungnam National University Hospital (CNUH IRB 2019-08-039), which uses a free data collection program (Vital recorder version 1.8, accessed at https://vitaldb.net, Seoul, Republic of Korea) [7].

Expired CO_2_ signal (capnography) and respiratory rate were monitored via anesthesia machine (Primus®, Dräger, Lübeck, Germany) and recorded at 62.5 and 0.25 Hz frequency, respectively. SpO_2_ signal was monitored with a disposable pulse oximetry sensor (Nellcor^TM^ Neonatal-Adult SpO_2_ sensor, Covidien, Mansfield, MA) and a patient monitor (Intellivue MX800, Philips, Boeblingen, Germany), with a recording frequency of 1 Hz.

### Data pre-processing

The collected data were extracted at a frequency of 1 Hz. To only include proper periods in the analysis, periods with respiratory rate (derived from anesthesia machine) and expired CO_2_ value over zero were selected. Then initial and last five minutes of data points were discarded to further exclude periods with error and noise signals due to patient positioning and/ or monitoring device arrangements.

### Outcome measures

Apnea event was defined as expired CO_2_<1 mmHg over consecutive 10 seconds [8]. Several parameters related to apnea event including incidence (per group), total apnea time, number of event (per case), apnea index (apnea event per hour), and time of the first apnea event (onset time) were assessed. Additionally, desaturation defined as SpO_2_<94% over consecutive 30 seconds was assessed [9].

### Apnea and desaturation detection algorithm

The apnea and desaturation were retrospectively assessed using a R program-based algorithm. The algorithm was internally validated by comparing the results with manual assessments (30 cases) performed by a blinded researcher (S.K.). The validation result is shown in S1 File.

### Statistical analysis

All statistical analyses were performed using R software version 4.2.2 (R Project for Statistical Computing, Vienna, Austria). Statistical sample size was not determined a priori. All feasible data during the study period were used. Missing values (patient weight and height) were addressed using ‘MICE’ package in R, which represents multivariate imputation by chined equation [10]. All variables handled in the current study were utilized as inputs for the imputation process. The first dataset resulting from this process was employed for the subsequent analysis. The distribution of continuous variables was assessed by the Shapiro–Wilk test, with normally distributed variables reported as mean ± SD and non-normally distributed variables as median (IQR). The p-values for pairwise comparisons between sedative agents were adjusted using Benjamini & Hochberg method. A p-value<0.05 was considered significant.

To address the selection bias of the sedatives used, we employed propensity score weighting using the ‘twang’ package in R [11]. This package uses gradient boosted models and allows for flexible approaches that automatically account for nonlinearities and interactions among the covariates. In the propensity score calculation model, we treated the sedative agent (i.e., group) as the dependent variable and included patient factors and clinical characteristics as covariates to estimate the population average treatment effect. The covariates were selected based on their clinical relevance and a comprehensive review of the existing literature on sleep apnea [12]. Specifically, we included comorbidities such as hypertension, diabetes, chronic kidney disease, and cerebrovascular disease, as they are known to be bidirectionally associated with sleep apnea [13]. Additionally, we considered demographic factors, including age, sex, and BMI, which are elements of the STOP-BANG questionnaire [14]. Furthermore, we accounted for other relevant intraoperative factors such as the patient’s position during surgery, type of anesthesia, use of supplementary drugs, and the duration of the surgical procedure.

All variables considered in propensity score calculation were included in the final logistic regression model with propensity score weighting to estimate the group effect on the apnea incidence (i.e., doubly robust estimation) [15]. A quasibinomial link function was used in the model to account for overdispersion. Multicollinearity was assessed by variance inflation factor and goodness-of-fit was evaluated using the Hosmer-Lemeshow test.

### Subgroup analysis

Univariable and multivariable logistic regressions were conducted to identify clinical factors associated with apnea occurrence in patients sedated with remimazolam. In addition to the aforementioned covariates, the dose of remimazolam was also included in the analysis. Variables with p-value<0.1 in the univariable analysis were included in the subsequent multivariable logistic regression.

## Results

**Fig 2:**
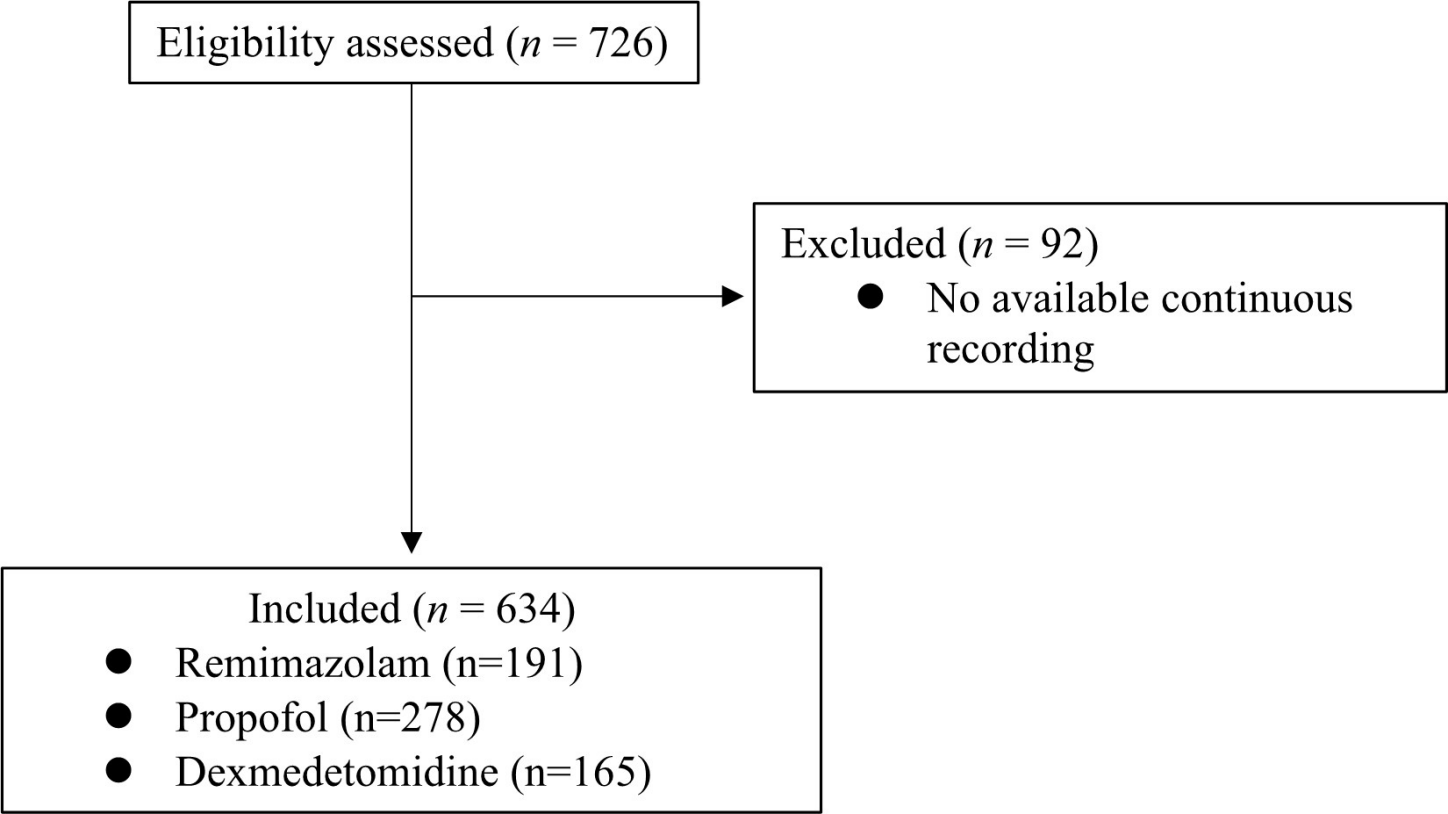
Patient flow diagram.

A total of 726 cases were assessed eligibility and 92 cases were excluded due to no available monitoring data and the remaining 634 cases were included in the final analysis (Fig 2). Nine and 12 missing values in patient weight and height were imputed. Clinical characteristics stratified by the agent used for sedation are summarized in Table 1. The doses (median, IQR) of sedatives used in group R, P, and D were 0.5 (0.4, 0.6) mg/kg/hr, 6.0 (4.5, 7.8) mg/kg/hr, and 0.9 (0.7, 1.2) μg/kg/hr, respectively.

**Table 1:** Clinical characteristics stratified by sedative agents.

	Remimazolam	Propofol	Dexmedetomidine	*p*
	(n = 191)	(n = 278)	(n = 165)	
Age (yr)	65.0	57.0	61.0	<0.001
	(54.0, 76.0)	(39.0, 66.0)	(46.0, 72.0)	
Sex (male)	79 (41.4)	138 (49.6)	79 (47.9)	0.197
Weight (kg)	64.7	64.0	63.5	0.226
	(54.0, 72.7)	(57.0, 73.8)	(54.5, 72.4)	
Height (cm)	159.0	163.4	161.3	<0.001
	(151.1, 166.6)	(156.0, 171.1)	(154.7, 171.8)	
BMI (kg/m^2^)	25.2	24.7	23.9	0.011
	(22.9, 27.7)	(22.2, 26.9)	(22.1, 26.7)	
ASA (>2)	50 (26.2)	43 (15.5)	39 (23.6)	0.011
HTN	94 (49.2)	82 (29.5)	60 (36.4)	<0.001
DM	45 (23.6)	37 (13.3)	38 (23.0)	0.006
CKD	14 (7.3)	7 (2.5)	10 (6.1)	0.043
CVD	11 (5.8)	13 (4.7)	10 (6.1)	0.788
Anesthesia type (spinal)	138 (72.3)	60 (21.6)	103 (62.4)	<0.001
Surgical position (supine)	163 (85.3)	258 (92.8)	129 (78.2)	<0.001
Surgery duration (hr)	1.7 (1.3, 2.4)	1.4 (1.1, 1.8)	1.7 (1.3, 2.3)	<0.001
Supplementary drug	15 (7.9)	19 (6.8)	51 (30.9)	<0.001
● Propofol (mg)	52.9 ± 23.6	NA	50.0 ± 29.2	0.855
● Midazolam (mg)	2.0 (1.0, 3.0)	2.0 (2.0, 2.5)	2.0 (2.0, 3.0)	0.907
● Fentanyl (μg)	30.0	30.0	30.0	0.616
	(20.0, 45.0)	(30.0, 45.0)	(20.0, 30.0)	

Values are median (IQR), mean ± SD, or number (%). Abbreviation: BMI, body mass index; ASA, American society of anesthesiologist physical status; HTN, hypertension; DM, diabetes mellitus; CKD, chronic kidney disease; CVD, cerebrovascular disease; NA, not available.

### Crude incidence

The apnea and desaturation events were summarized in Table 2. The incidence of apnea over 10 seconds was 63.9%, 67.3%, and 48.5% in group R, P, and D, respectively (overall, p<0.001). The incidence of desaturation over 30 seconds was 19.9%, 20.9%, and 10.9% in group R, P, and D, respectively (overall, p = 0.022).

**Table 2:** Apnea and desaturation events according to the sedative agents.

	Remimazolam (R)	Propofol (P)	Dexmedetomidine (D)	Overall *p*	D vs P	D vs R	R vs P
	(n = 191)	(n = 278)	(n = 165)		*p* ^*^	*p* ^*^	*p* ^*^
Apnea incidence (incidence per group)							
● 0	69 (36.1)	91 (32.7)	85 (51.5)	<0.001	<0.001	0.007	0.508
● 1–2	51 (26.7)	82 (29.5)	42 (25.5)	NA	NA	NA	NA
● >2	71 (37.2)	105 (37.8)	38 (23.0)	NA	NA	NA	NA
Total apnea time (seconds)	39.0 (0.0, 115.5)	40.5 (0.0, 185.0)	0.0 (0.0, 61.0)	<0.001	<0.001	0.104	0.018
Apnea event (per case)	1.0 (0.0, 4.0)	2.0 (0.0, 5.0)	0.0 (0.0, 2.0)	<0.001	0.011	0.194	0.173
Apnea index (event per hr)	1.0 (0.0, 3.3)	1.5 (0.0, 5.0)	0.0 (0.0, 1.7)	<0.001	<0.001	0.247	0.004
Apnea onset (min)	8.9 (4.6, 15.7)	9.5 (5.1, 18.8)	18.6 (10.5, 29.1)	<0.001	0.001	0.001	0.774
Desaturation event (incidence)	38 (19.9)	58 (20.9)	18 (10.9)	0.022	0.032	0.044	0.890

Values are number (%) or median (IQR). *Adjusted for the multiple comparisons. Abbreviations: NA, not available.

### Average treatment effect

The adjusted odds ratios (OR) of apnea were 2.33 (95% CI, 1.50 to 3.61) and 2.50 (95% CI, 1.63 to 3.85) in group R and P, respectively, compared to group D (Table 3). Supine position (OR 5.49, 95% CI 3.16 to 9.54), longer duration of surgery (hour; OR 2.20, 95% CI 1.62 to 2.97), and the use of supplementary drugs (OR 1.92, 95% CI 1.09 to 3.40) were found to be significant covariates. No collinearity or evidence of poor fit of the model was noted (p = 0.643). Detailed process of propensity score weighting is presented in S2 File.

**Table 3:** Propensity score weighted multivariable logistic regression model for the apnea incidence.

Characteristics	OR	95% CI	*p*
Sedative			
● Remimazolam	2.33	1.50 to 3.61	<0.001
● Propofol	2.50	1.63 to 3.85	<0.001
● Dexmedetomidine*	NA	NA	NA
Age (yr)	1.00	0.99 to 1.02	0.600
Sex (male)	1.25	0.85 to 1.82	0.252
BMI (kg/m^2^)	0.98	0.93 to 1.03	0.332
HTN	0.91	0.59 to 1.41	0.674
DM	1.11	0.68 to 1.82	0.669
CKD	2.23	0.89 to 5.59	0.089
CVD	2.34	0.96 to 5.72	0.063
Anesthesia type (spinal)	0.75	0.49 to 1.13	0.166
Surgical position (supine)	5.49	3.16 to 9.54	<0.001
Surgery duration (hr)	2.20	1.62 to 2.97	<0.001
Supplementary drug	1.92	1.09 to 3.40	0.025

*Dexmedetomidine as a reference group. Abbreviation: OR, odds ratio; CI, confidence interval; NA, not available; BMI, body mass index; HTN, hypertension; DM, diabetes mellitus; CKD, chronic kidney disease; CVD, cerebrovascular disease.

### Subgroup analysis (remimazolam)

The results of the subgroup analysis for group R are summarized in Table 4. The multivariable model also found to include supine position (OR 4.64, 95% CI 1.94 to 11.05) and longer duration of surgery (hour; OR 1.96, 95% CI 1.27 to 3.04) as significant covariates.

**Table 4:** Univariable and multivariable logistic regression for apnea incidence in patients sedated with remimazolam.

	Univariable			Multivariable		
Characteristics	OR	95% CI	*p*	OR	95% CI	*p*
Age (yr)	1.01	0.99 to 1.03	0.354			
Sex (male)	0.66	0.36 to 1.20	0.173			
BMI (kg/m^2^)	0.99	0.92 to 1.06	0.800			
HTN	1.31	0.72 to 2.37	0.373			
DM	1.53	0.74 to 3.17	0.250			
CKD	1.02	0.33 to 3.17	0.973			
CVD	2.67	0.56 to 12.72	0.218			
Anesthesia type (spinal)	1.23	0.64 to 2.36	0.533			
Surgical position (supine)	4.77	2.02 to 11.28	<0.001	4.64	1.94 to 11.05	<0.001
Surgery duration (hr)	1.93	1.28 to 2.92	0.002	1.96	1.26 to 3.04	0.003
Supplementary drug	1.61	0.49 to 5.26	0.431			
Remimazolam dose (mg/kg/hr)	0.81	0.27 to 2.47	0.712			

Abbreviation: OR, odds ratio; CI, confidence interval; BMI, body mass index; HTN, hypertension; DM, diabetes mellitus; CKD, chronic kidney disease; CVD, cerebrovascular disease.

## Discussion

In this study, we retrospectively assessed the occurrence of apnea during moderate to deep sedation induced by continuous infusion of remimazolam, and compared it with the commonly used agents, propofol or dexmedetomidine infusion. The regimen for remimazolam suggested in the current study showed apnea incidence over 60%, which is similar to that of group P and greater than that of group D. The propensity weighted multivariable analysis also verified this risk of apnea associated with the use remimazolam.

A recent randomized trial compared continuous infusion of remimazolam and dexmedetomidine in lower limb orthopedic surgery [16]. The study clearly showed differences in sedation characteristics between the two drugs which in turn highlights the challenge in comparison of sedative drugs. Despite attempts to target a specific level of sedation, deeper levels of sedation occurred more frequently in the remimazolam group. Considering the dosages used in that study, it is likely that deep sedation was more frequently achieved in the group R than group D in the current study.

The underlying mechanism of apneic events associated with remimazolam in this study might be mixed (central and obstructive) [17]. Nonetheless, based on our experience, most of the events are thought to be obstructive, given the fact that positive pressure ventilation was rarely needed. The importance of the supine position in the occurrence of apnea, as shown in the multivariable models, also suggests an obstructive nature of the apneic events. The association between supine position and obstructive sleep apnea is widely acknowledged [18]. Besides, midazolam along with propofol and dexmedetomidine is one of the commonly used drugs for inducing obstructive apnea for a diagnostic purpose (i.e., drug induced sleep endoscopy) [19].

The high incidence of the apneic events reported in this study is distinctive from previous studies. Considering the early onset of apnea (median 8.9 minute) in group R, it is highly likely that the loading dose is a major contributor to the event. However, the loading dose (0.1 mg/kg in 2 minutes) was not apparently high compared with previous studies (0.1 to 0.2 mg/kg for 1 minute or 5 mg) [20]. Therefore, other factors than mere dosage should be taken into consideration when comparing the respiratory safety profile of remimazolam reported in the current study and that from previous ones. First, most of the previous studies that reported low incidences of adverse respiratory events with the use of remimazolam were mainly done in endoscopic procedures [21–27]. This means that there would be at least some degree of discomfort that appeared during the endoscopic procedures even in the presence of pretreatments such as opioids and local anesthetics. Therefore, the sedative effect of remimazolam could have been more counterbalanced in the previous studies whereas it might have been more pronounced in the current study. Second, the position of the patient during the procedure should be considered. During typical endoscopic procedures, a lateral decubitus position is preferred. As supine position was found to be the strongest risk factor for the apneic event, the incidence of apnea could have been underestimated in procedures performed with lateral decubitus position (i.e., endoscopic procedures). Third, in the studies involving upper gastrointestinal endoscopy cases, the use of bite blockers and manipulation of the endoscopy itself might have affected the apneic event. Although it could not be revealed or proven during the retrospective analysis, most of the apneic events that occurred in the current study were obstructive pattern which can be readily interrupted by chin lifting, oral airway placement, and neck extension or rotation. These maneuvers can naturally occur during upper gastrointestinal endoscopic procedure itself.

This study tried to overcome the shortcomings of retrospective study by implementing several measures. Firstly, we utilized high resolution recording data of intraoperative signals, including capnography signal. This enabled us to develop a reliable apnea detection algorithm, which demonstrated higher fidelity than manual counting (see S1 File). Secondly, we took steps to minimize selection biases and group imbalances, allowing us to draw causal inferences using propensity score weighting and multivariable model, known as ‘doubly robust estimation’ [15]. However, it is important to note that since the study lacks detailed information on the actual depth of sedation achieved in each patient, it is more appropriate to appreciate the risk of apnea in relation to the specific sedation regimen used, rather than assuming equivalent depth of sedation between groups or attributing it solely to the drug characteristics itself.

While this study was able to implement several statistical measures to minimize potential biases, there are still some limitations that must be taken into consideration. Firstly, the threshold for intervention when an apneic event occurred was not predetermined and could be varied depending on the attending physician. This could introduce bias in outcomes such as total apnea time, apnea event per case, and desaturation event. However, it is important to note that bias in the overall incidence of apnea (whether it occurred or not) should be minimal, as no preventive measures were selectively applied in advance. Secondly, the level of sedation was not reported, so it is possible that there was some imbalance in the depth of sedation between the groups that could not be ruled out. Nonetheless, the sedations were in accordance with institutional protocol, implying that significant variations, especially at the initiation of each sedation, were unlikely. Additionally, the protocol aimed for at least a moderate degree of sedation.

Based on the findings, the authors propose the following considerations before the use of remimazolam for sedation during orthopedic surgery under regional anesthesia: 1) Continuous respiratory monitoring beyond SpO_2_, including expired CO_2_ monitoring, is strongly recommended; 2) Careful dosing is required, and if possible, avoid rapid dose escalation (e.g., large amount of loading or bolus doses); 3) Consider the duration and position of the procedure along with the accessibility for airway management during the procedure.

## Conclusions

The findings of the current study demonstrated a considerable incidence of apneic events during moderate to deep sedation with remimazolam infusion. Although these events did not result in severe adverse events, it is important not to underestimate the risk of apnea, and close monitoring is strongly advised.

## Supporting information

S1 File Summary of the algorithm verification process. (PDF)

S2 File Propensity score weighting. (PDF)
