# Supplementary material for: Apnea during moderate to deep sedation using continuous infusion of remimazolam compared to propofol and dexmedetomidine: A retrospective observational study
Source: PLoS One. 2024 Apr 17;19(4):e0301635. doi: 10.1371/journal.pone.0301635 (PMC11023199; doi:10.1371/journal.pone.0301635)
Supplement: S1 File — (PDF) [file pone.0301635.s001.pdf]

S1 file. Summary of the algorithm verification process

Process

1. A blinded researcher manually reviewed 30 randomly sampled vital records.

2. The following measurements were assessed:

- Total count of apnea events lasting over 10 seconds.
- Total count of apnea events lasting over 30 seconds.
- Total count of desaturation events (below 94%) lasting over 30 seconds.

3. The results of the manual assessment were compared to those generated by the algorithm.

4. Two-way mixed effect models were used to calculate the inter-rater correlation coefficient.

5. Lastly, binary outcomes (none or present) were derived from the count results and compared. Any discrepancies between the manual and algorithmic counts were reviewed to verify the accuracy of the algorithm.

Results

1. Total count of apnea events lasting over 10 seconds.

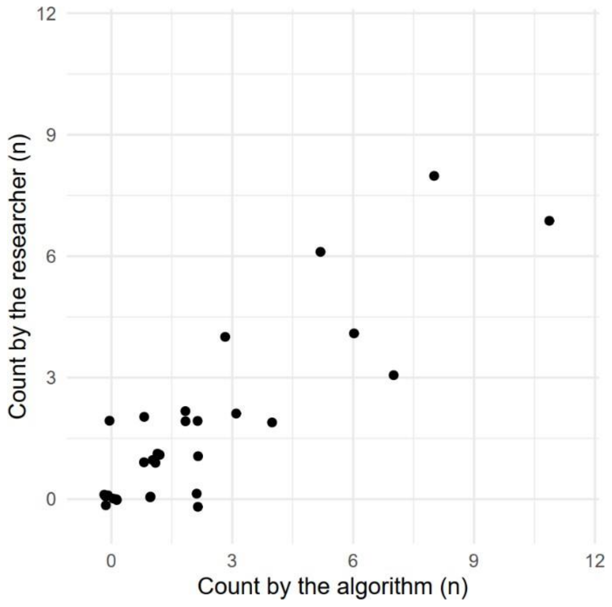

Inter-rater correlation: 0.851

(95% CI 0.710 to 0.926)

2. Total count of apnea events lasting over 30 seconds.

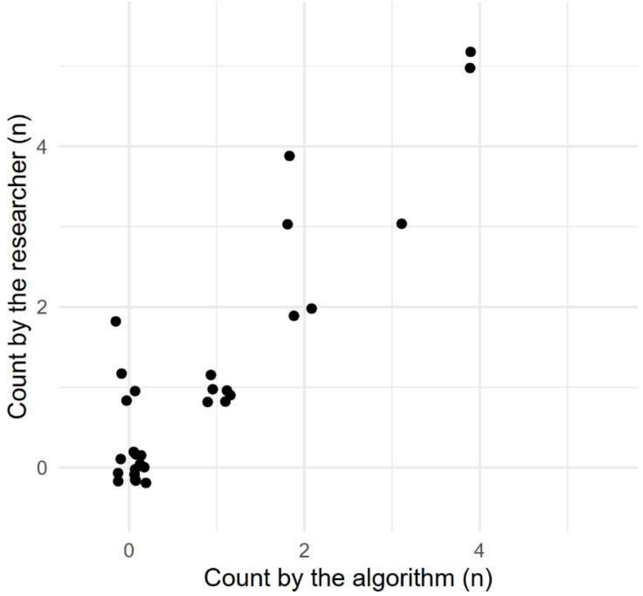

Inter-rater correlation: 0.900

(95% CI 0.800 to 0.951)

3. Total count of desaturation events (below 94%) lasting over 30 seconds.

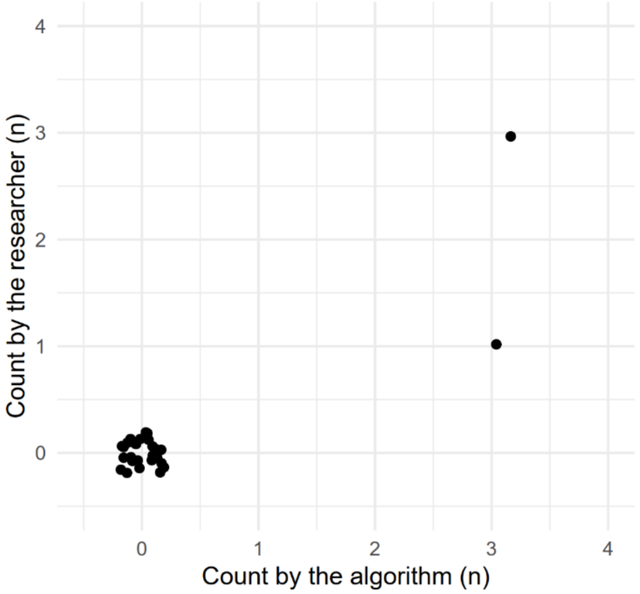

Inter-rater correlation: 0.853

(95% CI 0.714 to 0.927)

4. Occurrence of apnea > 10 seconds as a binary outcome (occur/ none)

		Researcher	
		None	Occur
Algorithm	None	7	1
	Occur	4	18

Five disagreements were noted, and it was verified that they were due to miscounting during the manual assessment.

5. Occurrence of apnea > 30 seconds as a binary outcome (occur/ none)

		Researcher	
		None	Occur
Algorithm	None	13	4
	Occur	0	13

Four disagreements were noted, and it was verified that they were due to miscounting during the manual assessment.

6. Occurrence of desaturation >30 seconds as a binary outcome (occur/ none)

		Researcher	
		None	Occur
Algorithm	None	28	0
	Occur	0	2

No disagreement was noted.
