# Supplementary material for: Apnea during moderate to deep sedation using continuous infusion of remimazolam compared to propofol and dexmedetomidine: A retrospective observational study
Source: PLoS One. 2024 Apr 17;19(4):e0301635. doi: 10.1371/journal.pone.0301635 (PMC11023199; doi:10.1371/journal.pone.0301635)
Supplement: S2 File — (PDF) [file pone.0301635.s002.pdf]

S2 file. Propensity score weighting

2023-08-06

Abbreviations and terms

wt: weight (kg)

ht: height (cm)

bmi: body mass index (kg/m2)

htn: hypertension

dm: diabetes mellitus

ckd_all: chronic kidney disease

cvd: cerebrovascular disease

ane_type: anesthesia type

BPB: brachial plexus block

Spinal: spinal anesthesia

position_1: surgical position

ane_duration_hr: surgery duration

supplementary_d: supplementary drug use

s_group: sedative group (D,P,R)

apnea_01: apnea (>10s; yes/no)

PS score calculation via mnps (multinomial propensity score) function

> ATE: average treatment effect on the population

```
mnps_mod_ATE <- mnps(s_group ~
                     age + sex + bmi +
                     htn + dm +  ckd_all + cvd +
                     ane_type + position_1 + supplementary_d + ane_duration_hr,
                     data = df2, estimand = "ATE", verbose = FALSE,
                     stop.method = c("es.mean","ks.max"), n.trees = 3000)
```

Check convergence of the PS model

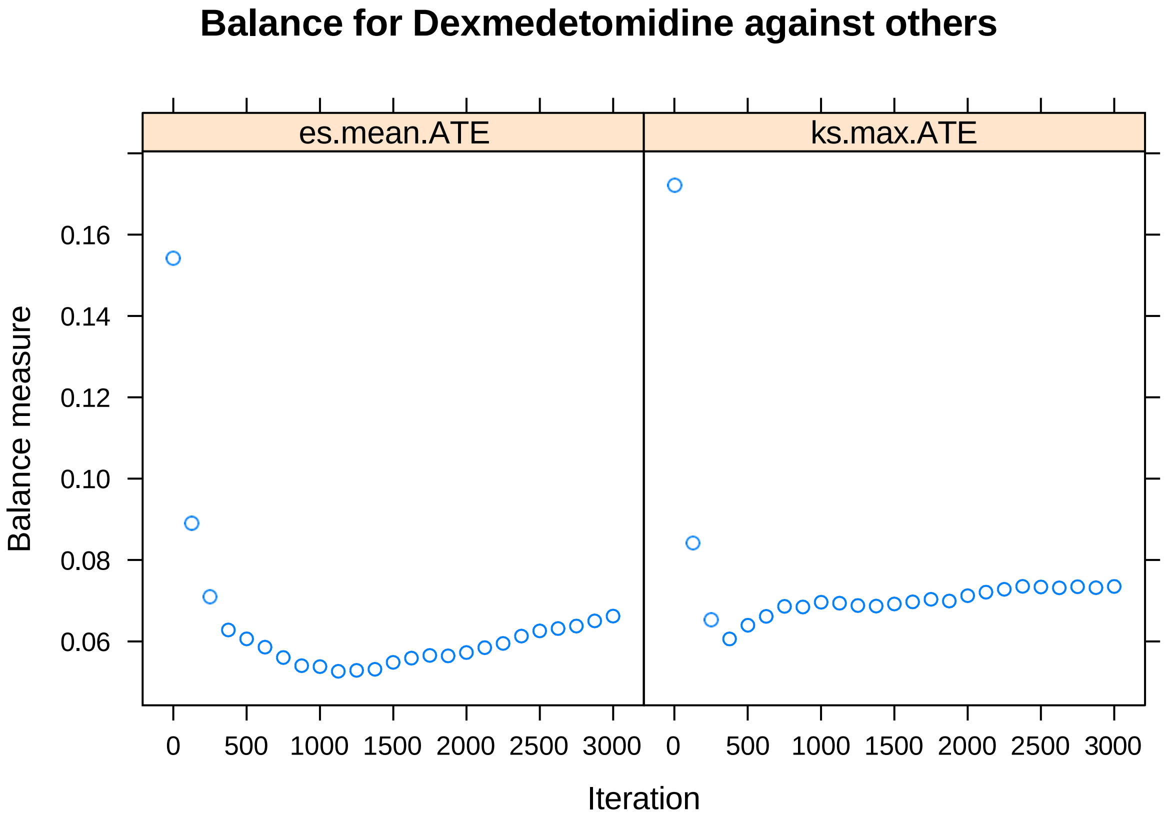

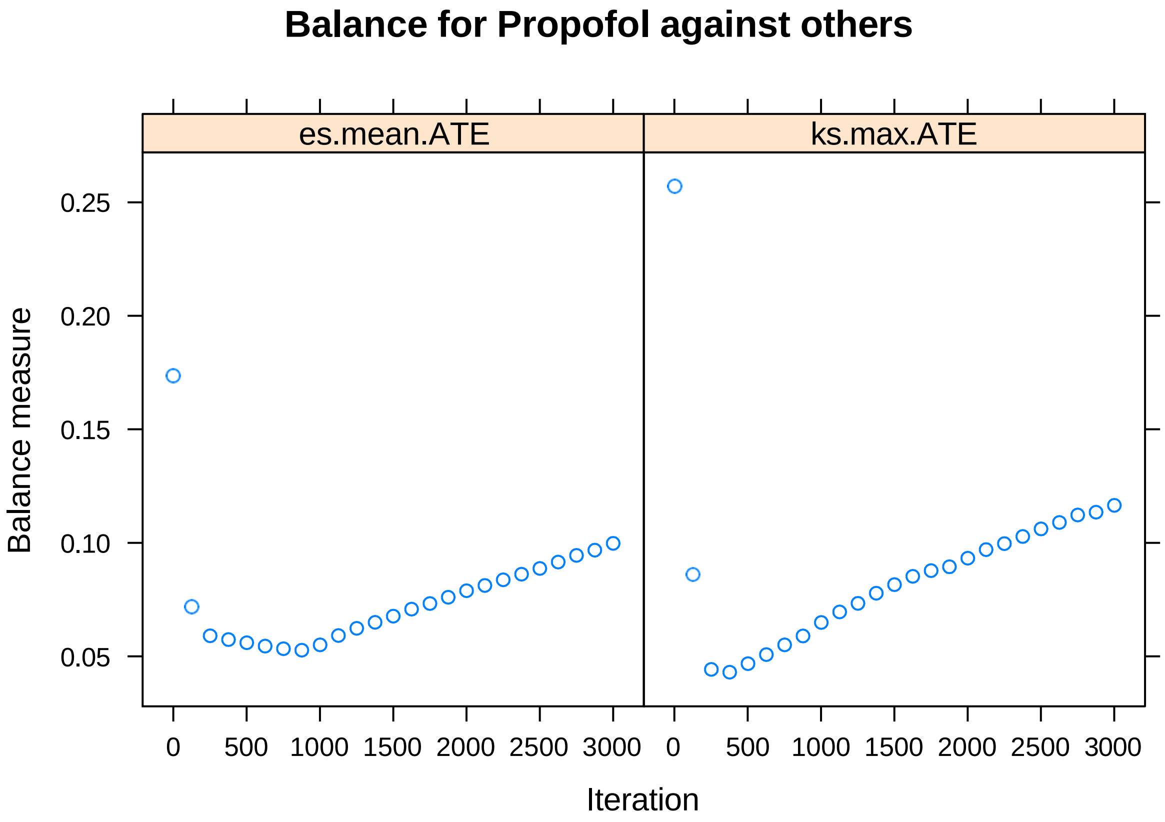

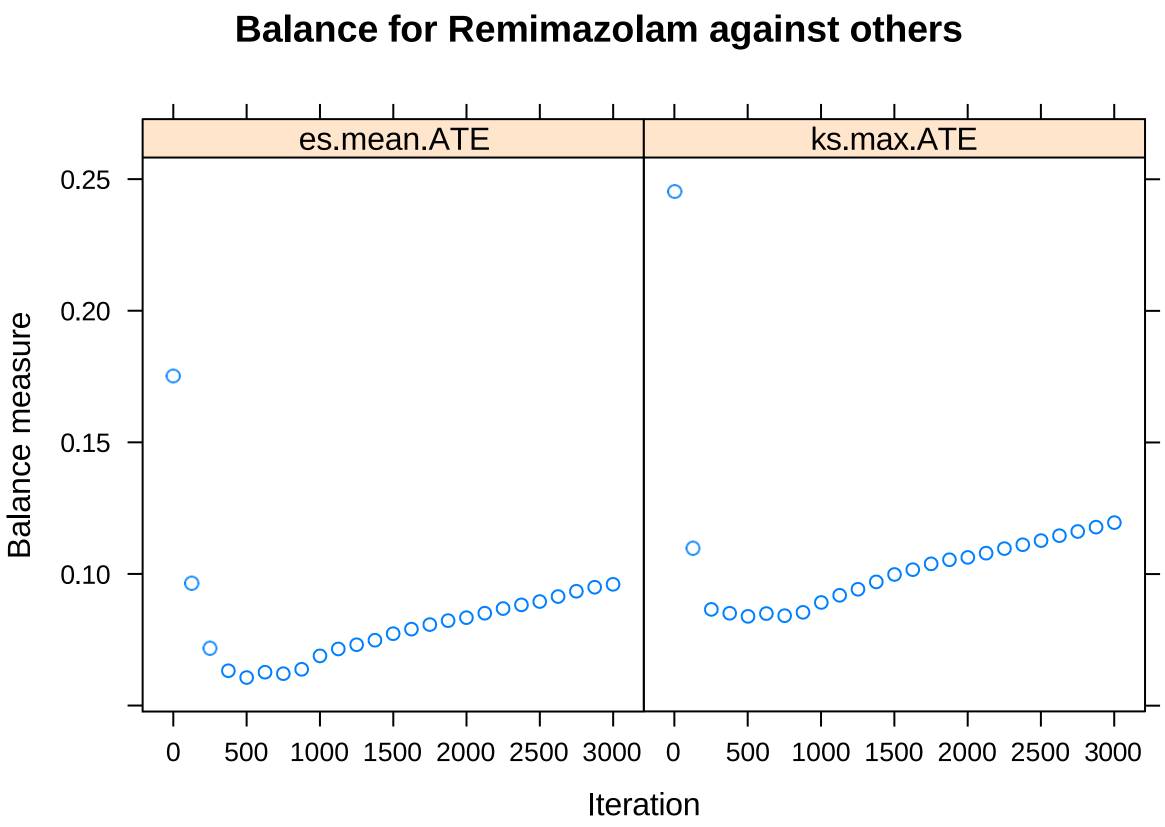
Balance for Remimazolam against others
es.mean.ATE
ks.max.ATE
Balance measure
0.25
0.20
0.15
0.10
0
500
1000
1500
2000
2500
3000
Iteration

```
## [[1]]
## NULL
```

> seems had enough interations (plateaued around 500-1000 iterations, and worsens thereafter)

Check overlap of PS distributions across the groups

Dexmedetomidine propensity scores by Tx group

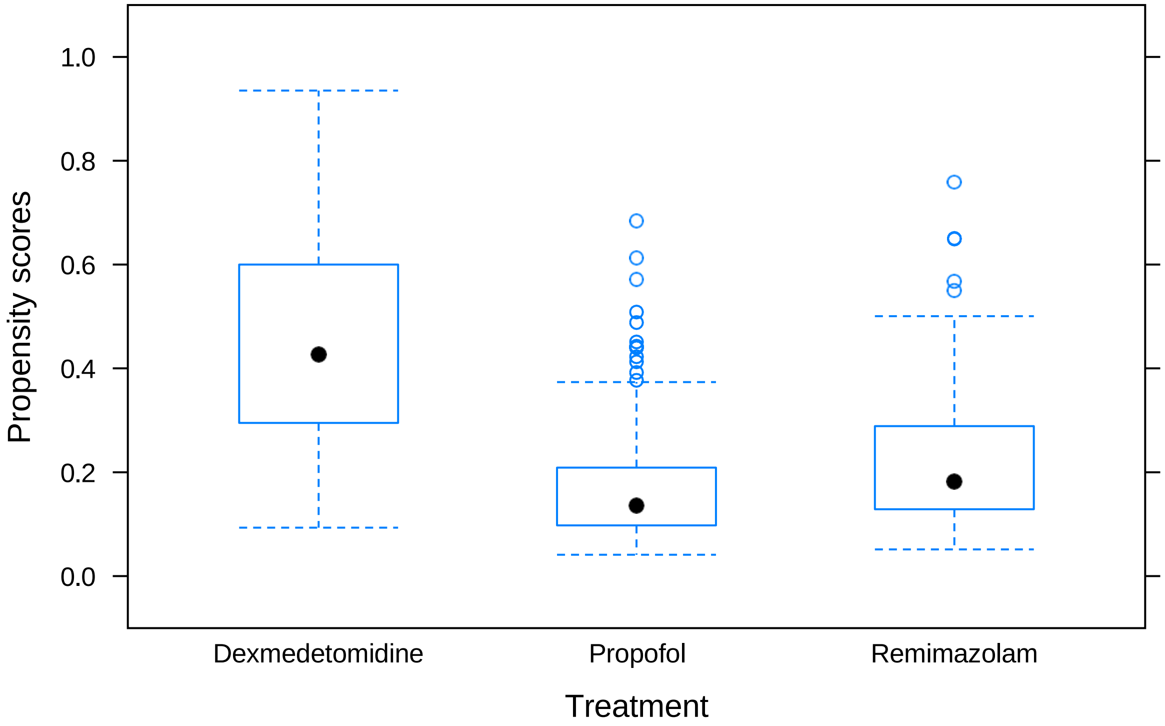

Propofol propensity scores by Tx group

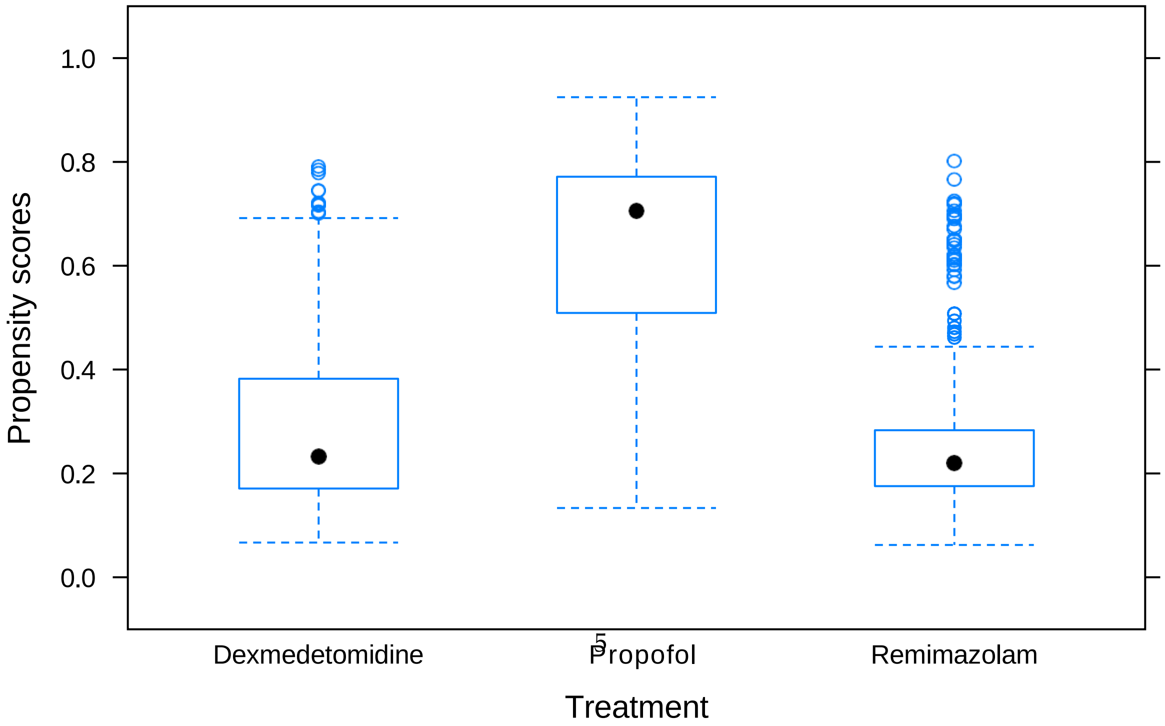

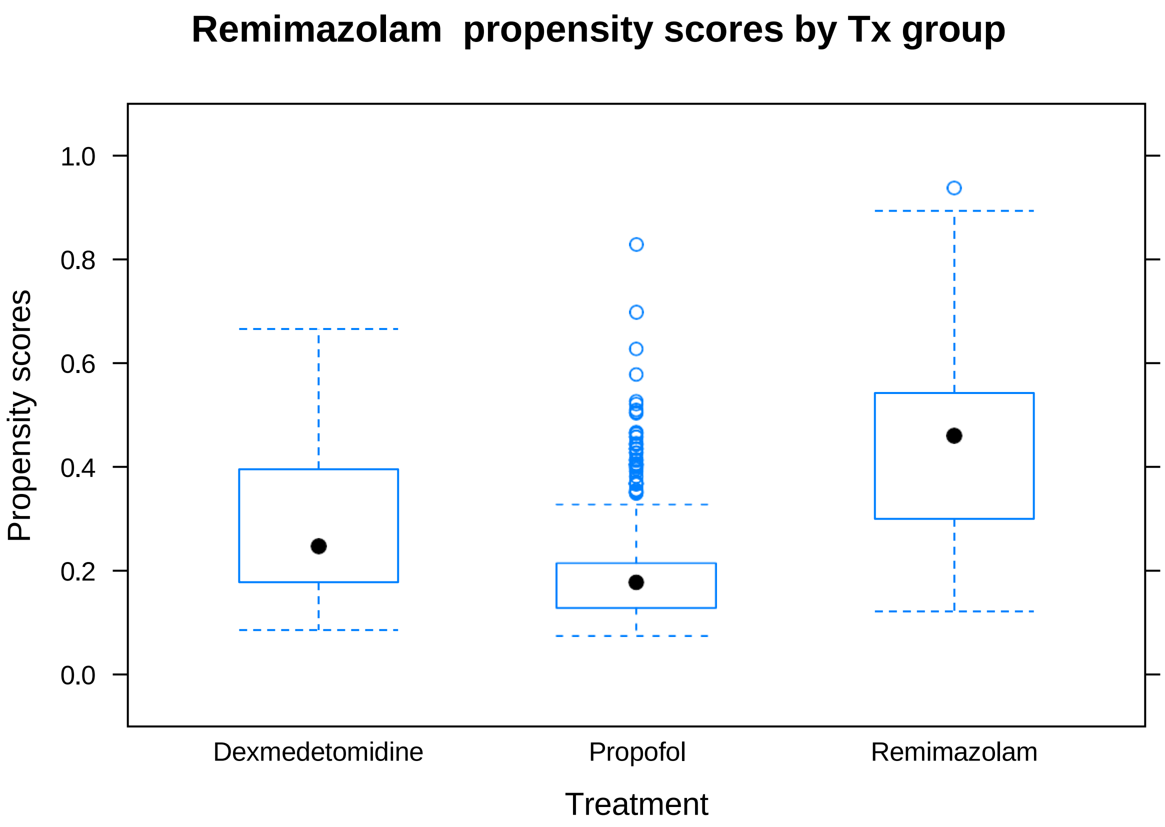
Remimazolam propensity scores by Tx group
Propensity scores
1.0
0.8
0.6
0.4
0.2
0.0
Dexmedetomidine
Propofol
Remimazolam
Treatment

```
## [[1]]
## NULL
```

Graphical assessments of balance before and after weighting

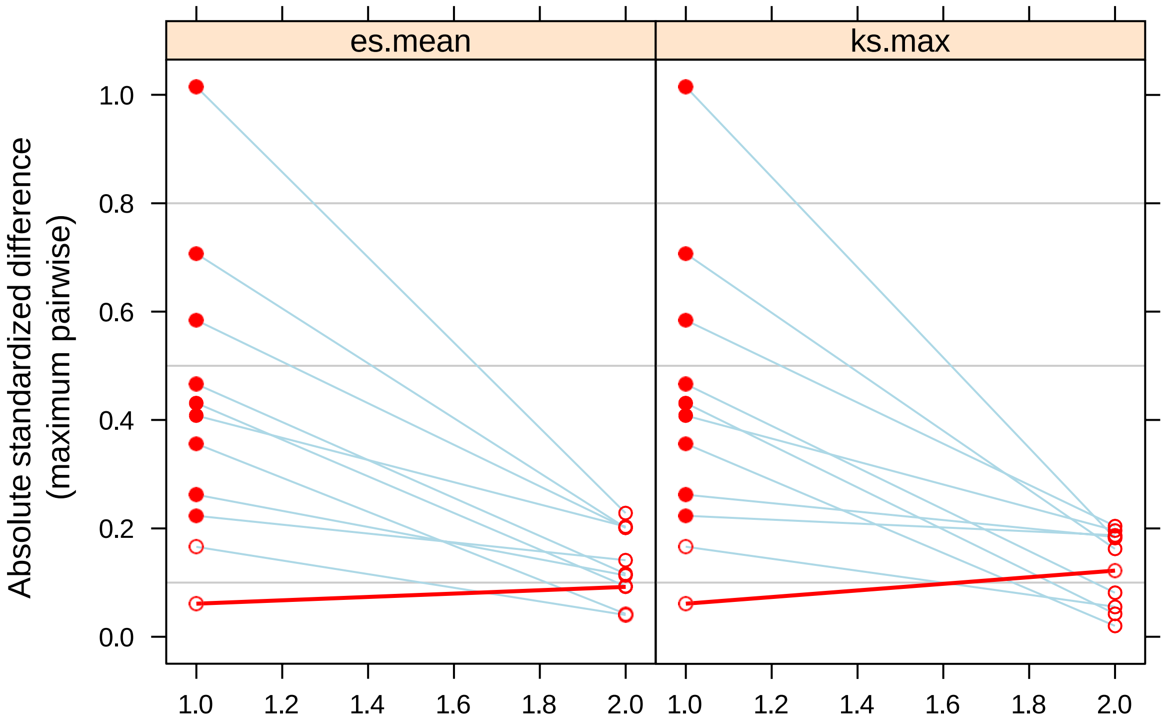

> Unblanced (large ASDs) before weighting (1.0 on X-axis)

> Much smaller ASDs after weighting (2.0 on X-axis)

> Dots indicate each covariate (therefore, 11 covariates in the PS model)

> Blue solid lines indicate decrease of ASD

> Red solid lines indicate increase of ASD

Check the increase of pairwise minimum p-values for differences between each of the individual treatment groups and observations in all other treatment groups

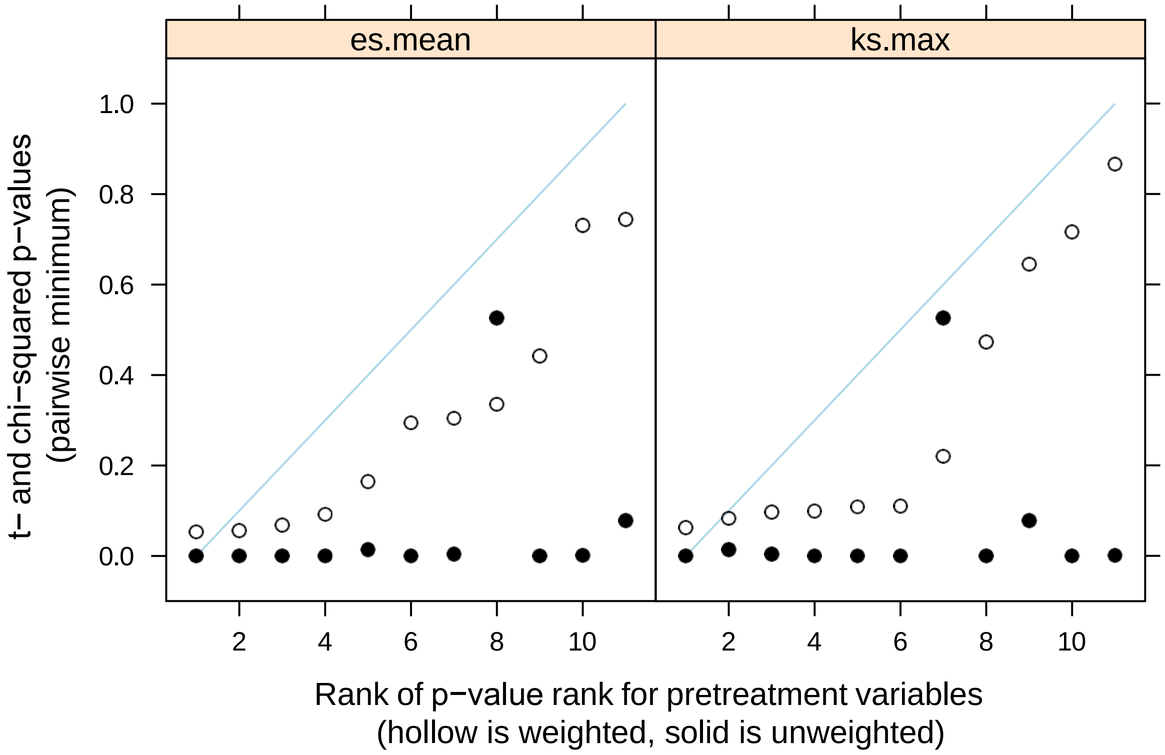

Check the increase of p values of KS test

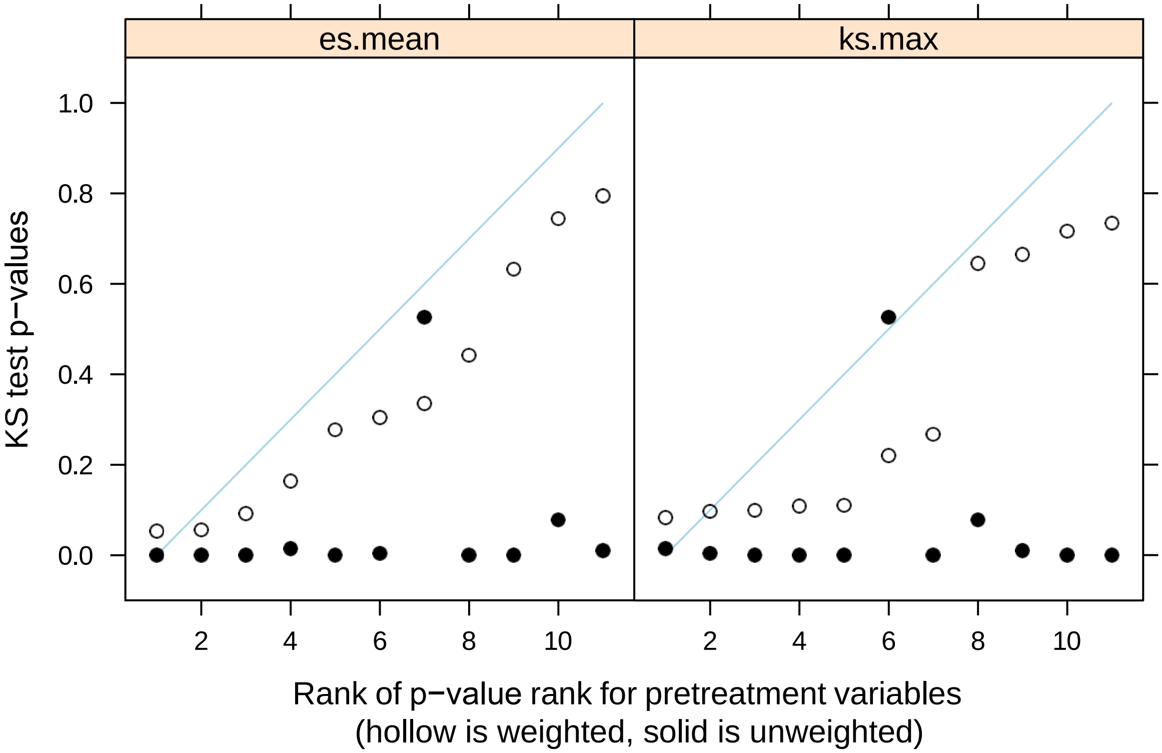

Check balance table

stop.method= unw : unweighted result

stop.method= es.mean / ks.max : weighted results

##		var	max.std.eff.sz	min.p	max.ks	min.ks.pval	stop.method
##	1	age	0.58	0.00	0.24	0.00	unw
##	2	sex:F	0.17	0.08	0.08	0.08	unw
##	4	bmi	0.36	0.00	0.17	0.01	unw
##	5	htn:0	0.41	0.00	0.20	0.00	unw
##	7	dm:0	0.26	0.00	0.10	0.00	unw
##	9	ckd_all:0	0.22	0.01	0.05	0.01	unw
##	11	cvd:0	0.06	0.53	0.01	0.53	unw
##	13	ane_type:BPB	1.01	0.00	0.51	0.00	unw
##	15	position_1:non-supine	0.43	0.00	0.15	0.00	unw
##	17	supplementary_d:no	0.71	0.00	0.24	0.00	unw
##	19	ane_duration_hr	0.47	0.00	0.21	0.00	unw
##	20	age	0.20	0.07	0.11	0.28	es.mean
##	21	sex:F	0.04	0.74	0.02	0.74	es.mean
##	23	bmi	0.04	0.73	0.08	0.79	es.mean
##	24	htn:0	0.20	0.09	0.10	0.09	es.mean
##	26	dm:0	0.11	0.30	0.04	0.30	es.mean
##	28	ckd_all:0	0.14	0.16	0.03	0.16	es.mean
##	30	cvd:0	0.09	0.34	0.02	0.34	es.mean
##	32	ane_type:BPB	0.23	0.05	0.11	0.05	es.mean
##	34	position_1:non-supine	0.09	0.44	0.03	0.44	es.mean
##	36	supplementary_d:no	0.20	0.06	0.07	0.06	es.mean
##	38	ane_duration_hr	0.12	0.29	0.09	0.63	es.mean
##	39	age	0.20	0.06	0.11	0.27	ks.max
##	40	sex:F	0.06	0.64	0.03	0.64	ks.max
##	42	bmi	0.02	0.87	0.09	0.66	ks.max
##	43	htn:0	0.20	0.10	0.09	0.10	ks.max
##	45	dm:0	0.18	0.10	0.07	0.10	ks.max
##	47	ckd_all:0	0.19	0.08	0.04	0.08	ks.max
##	49	cvd:0	0.12	0.22	0.03	0.22	ks.max
##	51	ane_type:BPB	0.18	0.11	0.09	0.11	ks.max
##	53	position_1:non-supine	0.04	0.72	0.01	0.72	ks.max
##	55	supplementary_d:no	0.16	0.11	0.06	0.11	ks.max
##	57	ane_duration_hr	0.08	0.47	0.08	0.73	ks.max

> Note the decrease of max.std.eff.sz from 'unw' to weighted results

> Note the increase of ks.pval from 'unw' to weighted results

Add weighting colunm on the dataset

'ks.max' was chosen based on larger effective sample size (ESS)

```
summary(mnps_mod_ATE)
```

```
## Summary of pairwise comparisons:
##   max.std.eff.sz       min.p    max.ks   min.ks.pval stop.method
## 1      1.0146648 8.015931e-25 0.5066858 8.015931e-25         unw
## 2      0.2281395 5.331329e-02 0.1139243 5.331329e-02     es.mean
## 3      0.2044478 6.170244e-02 0.1105430 8.296718e-02      ks.max
##
## Sample sizes and effective sample sizes:
##         treatment   n ESS.es.mean ESS:ks.max
## 1 Dexmedetomidine 165    124.6639   134.5011
## 2        Propofol 278    206.3526   211.9949
## 3     Remimazolam 191    144.6546   144.8756
```

```
df2$w <- get.weights(mnps_mod_ATE, stop.method = "ks.max")
```

Fully adjusted effect of group on apnea (>10 s) event

```
fit <- glm(apnea_01 ~ s_group +
                        age + sex + bmi +
                        htn + dm +  ckd_all + cvd +
                        ane_type + position_1 + supplementary_d + ane_duration_hr,
                        family = quasibinomial, data=df2, weights = w)
```

```
##
## Call:
## glm(formula = apnea_01 ~ s_group + age + sex + bmi + htn + dm +
##     ckd_all + cvd + ane_type + position_1 + supplementary_d +
##     ane_duration_hr, family = quasibinomial, data = df2, weights = w)
##
## Deviance Residuals:
##     Min       1Q   Median       3Q      Max
## -4.0427  -1.6723   0.9274   1.2629   4.8286
##
## Coefficients:
##                      Estimate Std. Error t value Pr(>|t|)
## (Intercept)         -2.714794   0.793177  -3.423 0.000661 ***
## s_groupPropofol      0.917329   0.219763   4.174 3.42e-05 ***
## s_groupRemimazolam   0.845845   0.224032   3.776 0.000175 ***
## age                  0.003176   0.006058   0.524 0.600269
## sexM                 0.221764   0.193492   1.146 0.252188
## bmi                 -0.024260   0.024984  -0.971 0.331904
## htn1                -0.094505   0.224429  -0.421 0.673835
## dm1                  0.107263   0.250867   0.428 0.669114
## ckd_all1             0.800671   0.469536   1.705 0.088651 .
## cvd1                 0.849742   0.456118   1.863 0.062937 .
## ane_typeSpinal      -0.294330   0.212020  -1.388 0.165568
## position_1supine     1.702561   0.282189   6.033 2.76e-09 ***
## supplementary_dyes   0.654556   0.290507   2.253 0.024598 *
## ane_duration_hr      0.787365   0.154339   5.102 4.48e-07 ***
## ---
## Signif. codes:  0 '***' 0.001 '**' 0.01 '*' 0.05 '.' 0.1 ' ' 1
##
## (Dispersion parameter for quasibinomial family taken to be 2.663336)
##
##     Null deviance: 2245.6  on 633  degrees of freedom
## Residual deviance: 1978.9  on 620  degrees of freedom
## AIC: NA
##
## Number of Fisher Scoring iterations: 4
```
